# Metabolic factors contribute to T‐cell inhibition in the ovarian cancer ascites

**DOI:** 10.1002/ijc.32990

**Published:** 2020-04-25

**Authors:** Yueqing Gong, Jianling Yang, Yan Wang, Lixiang Xue, Junjie Wang

**Affiliations:** ^1^ Center of Basic Medical Research Peking University Third Hospital Institute of Medical Innovation and Research Beijing China; ^2^ Biobank, Peking University Third Hospital Beijing China; ^3^ Medical Research Center Peking University Third Hospital Beijing China; ^4^ Department of Radiation Oncology Peking University Third Hospital Beijing China

**Keywords:** T‐cell, metabolism, immunosuppression, ascites, ovarian cancer

## Abstract

Malignant ascites is one of the major clinical features of ovarian cancer, which serves as a carrier for the peritoneal dissemination of tumor cells and predicts a poor prognosis in patients. In the microenvironment of ovarian cancer ascites, antitumor immunity is suppressed, which enables the tumor cells to escape from immune surveillance. The metabolic factors, including hypoxia, nutrient deprivation and accumulation of metabolic products, contribute to the immunosuppressive status of malignant ascites. The malignant ascites and ovarian solid tumors exhibit differential metabolic profiles. In this review, we have summarized the most recent findings on the interaction between immune cells and metabolic factors in the ovarian cancer ascites. The effects of metabolic factors on the antitumor functions of T‐cells in the malignant ascites were analyzed. Finally, we have discussed the potential directions for future research in this field.

**Abbreviations**
4‐HNE: 4‐hydroxy‐trans‐2‐nonenal
AA: arachidonic acid
ARG1: arginase‐1
CAFs: cancer‐associated fibroblasts
DCs: dendritic cells
DHA: docosahexaenoic acid
ER: endoplasmic reticulum
EVs: extracellular vesicles
FASN: fatty acid synthase
G1P: glucose‐1‐phosphate
HPLC: high‐performance liquid chromatography
IDO: indoleamine 2,3‐dioxygenase
IL‐2: interleukin‐2
iNOS: inducible nitric oxide synthase
LA: linoleic acid
LPA: lysophosphatidic acid
MDSCs: myeloid‐derived suppressor cells
MRI: magnetic resonance imaging
MSI: mass spectrometry imaging
PET: positron emission tomography
PGE2: prostaglandin E2
PPARs: peroxisome proliferators‐activated receptors
ROS: reactive oxygen species
TAMs: tumor‐associated macrophages
TDO: tryptophan 2,3‐dioxygenase
TLRs: toll‐like receptors
TME: tumor microenvironment
Tregs: regulatory T‐cells

## Introduction

Ascites is the abnormal accumulation of fluid in the abdominal cavity. Ascites caused due to malignancy is called malignant ascites.^1^ Carcinogenesis in the abdominal cavity is associated with the accumulation of fluids in the abdominal cavity, which is caused due to the exudation of tumor microvascular fluid and lymphatic obstruction.^2^ The accumulated fluid results in the formation of ascites. More than one‐third of patients with ovarian cancer exhibit ascites at diagnosis and almost all cases exhibit ascites at recurrence.^2^ The formation of malignant ascites, which is involved in the peritoneal dissemination of ovarian cancer, predicts a poor prognosis in patients with ovarian cancer.^2^ The malignant ascites contains cellular and noncellular components. The cellular components include the immune cells, stromal cells (such as cancer‐associated fibroblasts (CAFs), mesothelial cells and endothelial cells) and detached tumor cells, while the noncellular components comprise soluble factors and extracellular molecules, such as cytokines, chemokines, small molecular metabolites, cell‐free DNAs, RNAs, exosomes, microvesicles and extracellular matrix proteins.^2^ Malignant ascites and solid tumor tissue constitute the ovarian cancer microenvironment.

The suppressed antitumor immunity is one of the major characteristics of the malignant ascites microenvironment. The direct evidence of suppressed antitumor immunity is the immune cell profiles of the malignant ascites. In the ascites, the proportions of LAG‐3^+^, PD‐1^+^, TIM^+^ and CTLA‐4^+^ cells in both CD4^+^ and CD8^+^ cells were higher than those in the peripheral blood T‐cells.^3^ Several studies have demonstrated that the regulatory T‐cells (Tregs) and myeloid‐derived suppressor cells (MDSCs) accumulate in the ascites.^3^, ^4^, ^5^ Furthermore, *in vitro* experiments have demonstrated that the characteristics of cultured immune cells from normal tissue (e.g., peripheral blood from the normal specimen) change upon incubation with the cell‐free ovarian cancer ascites. For example, treatment with the malignant ascites suppresses the activity and proliferation of T‐cells and impairs their cytokine secretion ability.^6^, ^7^ Incubation with the malignant ascites can also attenuate the Toll‐like receptor (TLR)‐mediated activation of the dendritic cells (DCs).^8^ It is worth noting that the noncellular components in the ascites can affect the survival of immune cells and tumor cells. The ovarian cancer cells derived from the ascites are reported to constitutively secrete functional Fas ligand, which can induce apoptosis of the CD95/Fas‐positive immune cells.^9^ Some factors of the malignant ascites can activate the PI3K/AKT signaling pathway through αvβ5 integrin on the ovarian cancer cells and subsequently inhibit TRAIL‐induced apoptosis of tumor cells.^10^, ^11^ This is another mechanism for the tumor to escape the immune response.

Thus, the noncellular components in the ovarian cancer ascites are enough to exert immunosuppressive effects. Among these noncellular factors, the cytokines and chemokines are closely linked to the immune status of malignant ascites. Furthermore, several studies have suggested that metabolic factors contribute to the immunosuppressive status of malignant ascites. These metabolic factors in the microenvironment include hypoxia, nutrient depletion and accumulated immunosuppressive metabolites. These metabolic factors can directly affect the metabolism of various types of cells, including the immune cells.

In this review, we have summarized the metabolic factors in the ovarian cancer ascites and the effect of these factors on the antitumor functions of T‐cells. We have discussed the possible mechanism underlying the interactions between immune cells and metabolic factors. Ovarian cancer can be classified into the following five main histologic types: high‐grade serous carcinoma, low‐grade serous carcinoma, clear‐cell carcinoma, endometrioid carcinoma and mucinous carcinoma.^12^ This review mainly focuses on serous carcinoma, as more than 70% of ovarian cancer cases belong to serous carcinoma, which accounts for most deaths of ovarian cancer clinically.^13^

## Metabolic Factors Associated with T‐Cell Suppression in the Malignant Ascites

### Mild hypoxia

#### Oxygen content and pH value of the ovarian cancer ascites

The typical tumor microenvironment (TME) is characterized by hypoxia and low pH, which contribute to tumor growth and metastasis, as well as the suppression of antitumor immunity. In contrast to the hypoxic and acidic microenvironment of solid tumors, *the malignant ascites is hypoxic but not markedly acidic*. Previous studies have demonstrated that the O_2_ pressure in the patients with ovarian cancer is 45.65 ± 8.68 mm Hg in the malignant ascites.^14^ The O_2_ pressure in the healthy control subjects is approximately 100 mm Hg in the arterial vessel and approximately 40 mm Hg in the venous vessel.^15^ In the solid tumor, the O_2_ pressure decreases to 8–10 mm Hg.^15^ There are few studies that have reported the pH value of the ovarian cancer ascites. The preliminary results from our lab revealed that the pH value of the ovarian cancer ascites is >7.3. In contrast, the extracellular pH (pH_e_) values of the normal tissue and solid tumor tissue are approximately 7.4 and less than 7.0, respectively.^16^ As the malignant ascites is not markedly acidic, we will only discuss the immunosuppressive effects of hypoxia in the following sections.

#### Hypoxia mediates immunosuppression in most types of solid tumor

Hypoxia may stimulate the immune cells in some cases. However, *hypoxia is a key factor that mediates immunosuppression in a typical solid tumor microenvironment*.^17^ Hypoxia directly suppresses antitumor immunity by suppressing the proliferation, differentiation, survival and function of the T‐cells. For example, the survival rate of T‐cells markedly decreases at low O_2_ levels,^18^ and the interleukin‐2 (IL‐2) secretion by naïve T‐cells decreases as HIF1α becomes more stable under hypoxic conditions.^19^ In addition, hypoxia can indirectly affect the T‐cell function. For example, the hypoxic condition promotes tumor cells to produce immunosuppressive factors, such as TGFβ, IL‐10, VEGF, IL‐6, CCL22, CCL28 and galectin‐1/3.^17^ These factors can not only directly suppress T‐cell function but also promote the recruitment and polarization of immunosuppressive cells, such as MDSCs and tumor‐associated macrophages (TAMs).^20^ Hypoxia also promotes the expression of PD‐L1 on the tumor cells and MDSCs, which suppresses T‐cell function.^21^, ^22^

#### Effects of hypoxia in solid ovarian cancer tissue on the immune status of malignant ascites

Compared to the peripheral blood, the ascitic fluid is more hypoxic. HIF1α is the master regulator that regulates the cellular response to hypoxia. It functions as a transcriptional factor, which activates over 40 genes associated with metabolic adaptation to hypoxia. Previous studies have demonstrated that the stability of HIF1α markedly increases at O_2_ pressure of 2 kPa (~15 mm Hg) and below, which activates the hypoxic response.^23^ However, the O_2_ level in the ovarian cancer ascites is above 2 kPa. In contrast, HIF2α can be stabilized under a relatively mild hypoxic condition (O_2_ level is lower than 5%).^24^ Therefore, HIF2α may be activated under hypoxic conditions in the malignant ascites. The downstream effects of HIF1α and HIF2α activation are different.^24^, ^25^, ^26^ Hence, further studies are needed to identify the role of mild hypoxia in mediating immunosuppression in the ascites.

##### Hypoxia in the solid tumor tissue of ovarian cancer may indirectly contribute to immunosuppression in the malignant ascites

**Figure 1:**
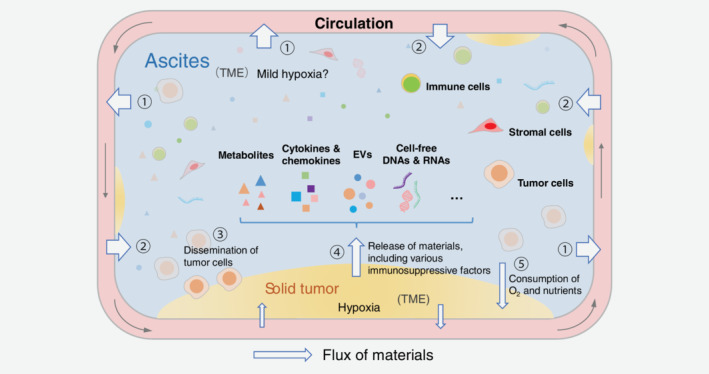
Unique microenvironment of ovarian cancer in the abdominal cavity. The malignant ascites and solid tumor tissue constitute the ovarian cancer microenvironment, which can be regarded as an evolutionary outcome of the original normal tissue microenvironment in the abdominal cavity. There is a frequent material exchange in this microenvironment. The ascites is continuously exchanging materials with the circulatory system (marked with ① and ②), and the solid tumor tissue (marked with ③, ④ and ⑤). The solid tumor tissue can release various soluble immunosuppressive factors into the ascites fluid and may consume the O_2_ and nutrients in the ascites. These effects may contribute to converting the microenvironment of normal tissue into the tumor microenvironment. However, these effects are “diluted” due to the material exchange between ascites and peripheral blood. The O_2_ and nutrients can be replenished from the peripheral blood, and the immunosuppressive factors may diffuse out into the blood. Even so, the diluted soluble factors in the malignant ascites can still mediate immunosuppression effectively. The ascites microenvironment is unique when compared to the normal tissue and typical tumor microenvironment (TME) of the solid tumors.

Several studies have demonstrated that the solid tumor tissue of ovarian cancer exhibits an enhanced expression of HIF1α,^27^ which mediates a series of immunosuppressive effects.^28^, ^29^, ^30^ The close proximity between solid tumor tissue in the abdominal cavity and malignant ascites enables the exchange of various factors between them. Thus, various hypoxia‐induced immunosuppressive factors in the solid tumor tissue may be secreted into the ascites (Fig. 1). For example, hypoxia in the solid tumor microenvironment can upregulate the expression of CCL28, which contributes to the recruitment of Tregs, in a mouse model of ovarian cancer.^28^ The overexpression of CCL28 in the solid tumor tissue also enhances the level of CCL28 in the ascites.^28^

##### Hypoxia can induce the production of various immunosuppressive metabolic factors

**Figure 2:**
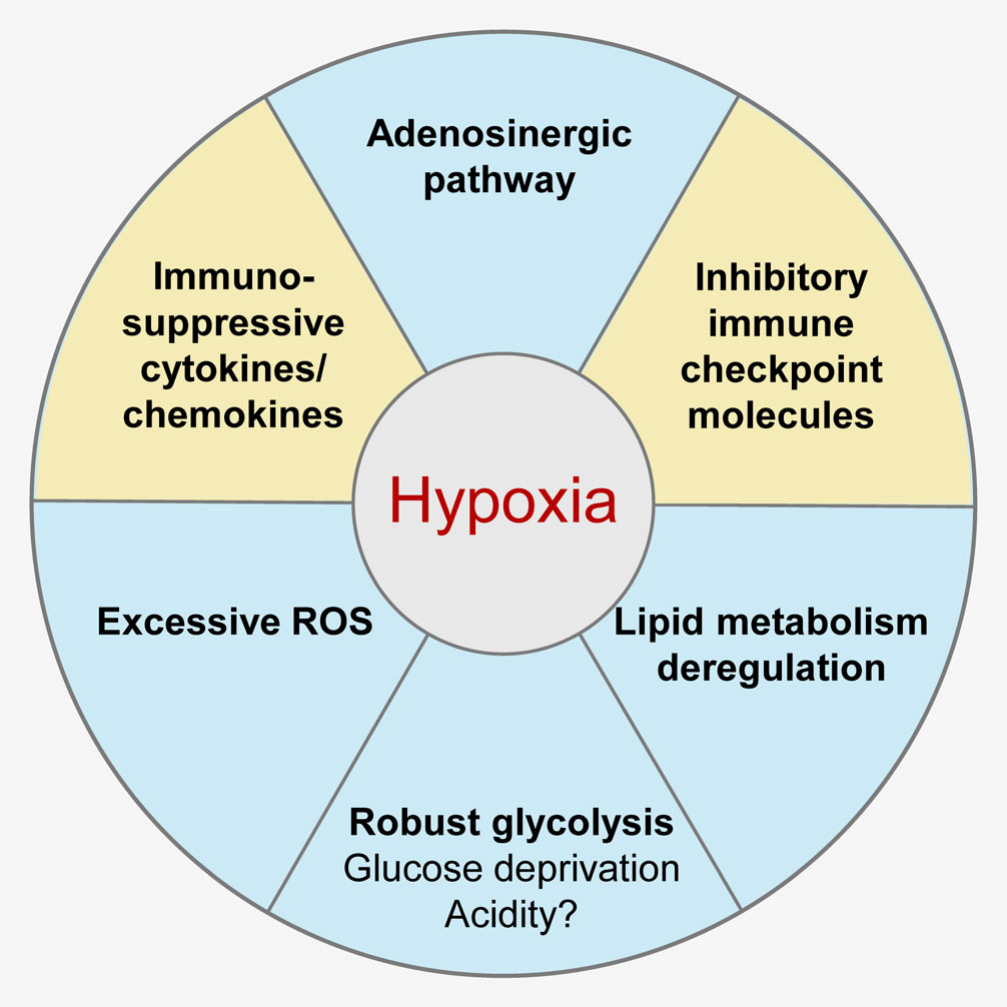
Hypoxia is a key factor in mediating immunosuppression in ovarian cancer. Hypoxia can directly suppress T‐cell function and induce the production of various immunosuppressive factors, such as TGFβ and IL‐10. Hypoxia can also promote the expression of inhibitory immune checkpoint molecules, such as PD‐L1. Additionally, hypoxia can strengthen other immunosuppressive metabolic factors, including reactive oxygen species (ROS) and adenosine. Hypoxia can also promote the expression of COX‐2, which enhances the synthesis of PGE2.

These immunosuppressive metabolic factors have been found in the ovarian cancer ascites. For example, hypoxia can increase the production of prostaglandin E2 (PGE2),^31^ reactive oxygen species (ROS)^32^ and adenosine.^33^ These factors contribute to T‐cell inhibition in the malignant ascites (discussed in the following sections). Thus, hypoxia may play a central role in regulating the metabolic factors that can mediate immunosuppression in the ascites (Fig. 2).

### Deficiency of nutrients

Similar to the tumor cells, the activated T‐cells require a high amount of nutrients, such as glucose and amino acids to drive protein synthesis and glycolysis (Warburg effect) during proliferation.^34^, ^35^ Additionally, the tumor cells can promote glycolysis in the stromal cells, such as CAFs and mesothelial cells, which involves high consumption of glucose and other nutrients in the TME.^36^, ^37^ Consequently, the competition among tumor cells, tumor‐associated stromal cells and T‐cells leads to nutrient deficiency in the T‐cells, which can impair the antitumor function of T‐cells.^38^, ^39^

#### Glucose deprivation

##### Glucose is an essential nutrient for the proliferation of effector T‐cells

Glucose deficiency directly suppresses the activation and proliferation of T‐cells and induces T‐cell anergy and death.^40^, ^41^, ^42^ In contrast to the effector T‐cells, the Tregs exhibit downregulated glycolysis and rely mainly on the oxidation of fatty acids for energy.^43^, ^44^ Glucose deficiency promotes the induction of Tregs.^43^

##### Glucose is actively consumed in the ovarian cancer ascites but is not depleted

Previous studies have demonstrated that the ascites from patients with ovarian cancer exhibits a lower level of glucose and a higher level of glucose‐1‐phosphate (G1P) than the ascites from patients with liver cirrhosis.^45^ This suggested an enhanced consumption of glucose in the malignant ascites. However, the glucose level in the malignant ascites was similar to the normal blood glucose levels.^6^ The blood capillaries are rich in the peritoneum, which is a semipermeable membrane.^46^ Hence, glucose in the malignant ascites may be replenished from the peripheral blood through the peritoneum (Fig. 1). Therefore, enhanced glucose consumption in the ovarian cancer ascites may not result in glucose deficiency in the T‐cells.

However, *the ovarian cancer ascites can hamper the glucose uptake by T‐cells, which impairs the T‐cell immune function*. GLUT1 is the key transporter in the plasma membrane for T‐cell glucose uptake.^47^ The cell‐free malignant ascites can markedly decrease the expression of GLUT1 and subsequently downregulate glycolysis.^6^ The decreased glucose uptake also results in the inhibition of N‐linked protein glycosylation, which induces endoplasmic reticulum (ER) stress. ER stress triggers the IRE1α‐XBP1 pathway and contributes to the abnormal mitochondrial activity of aerobic respiration and glutamine intake. The activities of CD4^+^ T‐cells markedly decline upon incubation with the malignant ascites. The animal studies demonstrated that blocking the IRE1α‐XBP1 pathway can rescue the malignant ascites‐induced attenuated antitumor activity of T‐cells.^6^ Further investigations are needed to determine the mechanism underlying the suppression of GLUT1 expression in the T‐cells by the malignant ascites.

#### Amino acid catabolism

Generally, amino acid catabolism contributes to immunosuppression in the TME. The tumor cells and immunosuppressive cells (such as MDSCs and TAMs) consume high amounts of amino acids and produce various metabolic products.^35^, ^48^ Both amino acid depletion and products of amino acid catabolism can directly suppress the antitumor functions of T‐cells.^35^ *Tryptophan and arginine catabolism are the hallmarks of TME metabolism*. Indoleamine 2,3‐dioxygenase (IDO) and tryptophan 2,3‐dioxygenase (TDO) are rate‐limiting enzymes during tryptophan decomposition. Arginase‐1 (ARG1) and inducible nitric oxide synthase (iNOS) are key enzymes involved in arginine catabolism. These enzymes can mediate immunosuppressive effects,^35^ and they are primarily located within the cells.^49^ In addition, studies have shown that ARG1 can be released into the extracellular space, and keep its immunosuppressive activity.^50^, ^51^

Previous studies have demonstrated that IDO1, ARG1 and iNOS are expressed in various types of cells in the ovarian cancer ascites (Table 1). Additionally, the CD14^+^HLA‐DR^−/low^ MDSCs cultured with the ascites from patients with ovarian cancer *in vitro* exhibited upregulated expression of ARG1 and iNOS.^53^ Treatment with inhibitors of IDO1, ARG1 or iNOS, or with L‐arginine abolished the suppressive effects of MDSCs derived from/induced by ascites on T‐cells.^52^, ^53^ Interestingly, the extracellular vesicles (EVs) in the ovarian cancer ascites also exhibit ARG1 expression.^51^ *In vitro* studies have demonstrated that EVs containing ARG1 can inhibit T‐cell proliferation, which can be mitigated by ARG1 inhibitor or l‐arginine. These EVs can also be internalized by DCs, which blocks the DC‐primed T‐cell proliferation.^51^ Thus, IDO1, ARG1 and iNOS are involved in T‐cell inhibition in the ovarian cancer ascites.

**Table 1:** An overview of IDO1/ARG1/iNOS expression status associated with human ovarian ascites

Cell types	Enzymes of amino acid catabolism	Usage of enzyme inhibitors in the reports	References
MDSC derived from ascites	IDO1/ARG1/NOS	1‐MT (IDO inhibitor) Nor‐NOHA (arginase inhibitor) L‐NMMA (NOS inhibitor)	52
MDSC (CD14^+^HLA‐DR^−/low^) induced by ascites	ARG1/iNOS	Nor‐NOHA (arginase inhibitor) SMT (iNOS inhibitor)	53
MDSC derived from ascites	IDO1/ARG1	N/A	54
MDSC (CD33^+^) derived from ascites	ARG1	N/A	55
Macrophage derived from ascites	ARG1	N/A	56
Macrophage derived from ascites	ARG1	N/A	57
Ascites‐derived tumor cells and extracellular vesicles	ARG1	OAT‐1746 (arginase inhibitor)	51

### Immunosuppressive metabolic products

In addition to lactate and the products of amino acid catabolism, other metabolic products, such as lipids, adenosine and ROS also accumulate in the malignant ascites. These metabolic products can also affect T‐cell function.

#### Immunosuppressive functions of lipids in the ovarian cancer ascites

##### Origin of lipids in the ovarian cancer ascites

The metabolomic analysis revealed that the ovarian cancer ascites contains different types of lipids, including saturated and unsaturated fatty acids, fatty acid amides, cholesterol and its derivatives, as well as various forms of phospholipids.^45^, ^58^ Lipid accumulation is one of the major characteristics of the ovarian cancer ascites. These lipids may be derived from the adipose cells,^59^ which are rich in omentum, and tumor cells.^60^ The omental adipocytes are reported to supply fatty acids to the tumor cells *via* a FABP4‐dependent mechanism to promote homing, migration and invasion of tumor cells.^59^ Additionally, the human ovarian cancer tissue exhibits higher fatty acid synthase (FASN) expression than the normal ovarian tissue, and the expression level of FASN in the metastatic tumor is higher than that in the primary tumor.^58^, ^60^ In the mouse model of ovarian cancer, the upregulation of FASN in the tumor cells results in an elevated level of lipids in the ascites. In the ascites of ID8 tumor‐bearing mice, the level of saturated and unsaturated fatty acids, as well as the level of triglyceride, is positively correlated with the expression level of FASN.^60^

##### Abundant lipids induce robust lipid metabolism in the ovarian cancer ascites

The lipids enriched in the ovarian cancer microenvironment may cause global changes in lipid metabolism.^58^ The tumor cells and immune cells have multiple lipid‐sensing pathways, which regulate lipid metabolism and other cellular processes. The ovarian cancer ascites contain various types of unsaturated fatty acids, including linoleic acid (LA), arachidonic acid (AA) and docosahexaenoic acid (DHA).^45^, ^61^ The concentrations of LA, AA and DHA are above the half‐maximal inhibitory concentration (IC50) required for binding to the peroxisome proliferators‐activated receptors (PPARs), which are a group of nuclear receptor proteins, and play key roles in fatty acid sensing.^61^, ^62^, ^63^ The functions of activated PPARs vary between cell and tissue types. On the whole, the activated PPARs not only upregulate the levels of lipid synthesis and storage, but also shift the level of lipid degradation and oxidation.^62^ Several studies have reported that the level of ketone bodies in the ovarian cancer ascites was higher than that in the effusions of breast carcinoma and mesothelioma.^64^ This indicates robust lipid degradation in the TME of ovarian cancer.

##### Robust lipid metabolism in the ovarian cancer ascites suppresses the antitumor functions of T‐cell through multiple pathways

The unsaturated fatty acids regulate immune functions via PPARs. PPARs can be activated by the enriched unsaturated fatty acids in the ovarian cancer ascites. However, there are contradictory reports on the mechanism underlying the regulation of PPARs. Some studies have reported that PPARγ is involved in the suppression of T‐cell function by inhibiting IL‐2 production directly.^65^ However, other studies have reported that PPAR‐induced fatty acid oxidation in T‐cells increases the number of active effector CD8^+^ T‐cells and subsequently facilitates anti‐PD‐1 therapy.^66^ Moreover, unsaturated fatty acids in the malignant ascites can also regulate the functions of macrophages *via* PPARs, which may indirectly affect the T‐cell function. Past research has demonstrated that PPARδ/β in the macrophage is activated by LA, AA and DHA in the ovarian cancer ascites. Thus, the gene expression patterns are affected in the macrophages, which contribute to the protumorigenic polarization of TAMs.^61^

Some types of unsaturated fatty acids in the ovarian cancer ascites can be converted into eicosanoids, which regulate antitumor immunity. For example, LA and AA in the malignant ascites can be transformed into PGE2 by COX‐2,^5^ which is a kind of cyclooxygenase actively expressed in various types of cells (such as MDSCs). In the TME, PGE2 can mediate T‐cell inhibition indirectly through multiple pathways.^52^, ^67^ In the ovarian cancer ascites, PGE2 can induce the secretion of CXCL12 and the expression of CXCR4 to promote MDSC accumulation, which inhibits T‐cell functions.^5^ Hypoxia, a characteristic of ovarian cancer TME, can upregulate the expression of COX‐2 and subsequently promote the synthesis of PGE2.^31^

The byproducts of lipid metabolism can regulate antitumor immunity in the ovarian cancer ascites. The accumulation of lipid peroxide is often observed in the dysfunctional DCs in the TME.^68^, ^69^ The enhanced level of ROS in the ovarian cancer ascites can cause lipid peroxidation in the DCs and generate reactive byproducts, such as 4‐hydroxy‐trans‐2‐nonenal (4‐HNE).^70^ Previous studies have demonstrated that 4‐HNE can form stable adducts with the ER‐resident chaperones in these DCs and subsequently trigger ER stress. The ER stress activates the IRE1α‐XBP1 pathway and upregulates various triglyceride biosynthetic genes. *In vitro* experiments have revealed that the lipid levels in DCs markedly increase upon incubation with the cell‐free malignant ascites. Additionally, these DCs exhibit impaired antigen‐presenting capacity, which affects the T‐cell activation.^70^ The lipid accumulation is suppressed and the number of lipid droplets decreases when XBP1 is knocked out in the DCs. XBP1 knockout enhances the ability of DCs to activate the T‐cells, suppresses the tumor growth and prolongs the mouse lifespan.^70^ Additionally, lipid accumulation occurs in the macrophages of the ovarian cancer ascites.^61^ Some studies suggest that these lipids may be derived from the extracellular environment.^61^ Thus, both lipid synthesis and uptake can cause lipid accumulation in certain types of immune cells and consequently remodel their immune function.

Phospholipids are also detected in the malignant ascites.^45^ Lysophosphatidic acid (LPA) is closely associated with ovarian cancer progression, which may cause excessive cell proliferation and result in carcinogenesis and metastasis. A considerable amount of LPA in the ovarian cancer ascites is obtained from the peritoneal mesothelial cells.^71^ Studies on a mouse melanoma model have demonstrated that LPA can suppress the activation and proliferation of CD8^+^ T‐cells.^72^ However, the effects of LPA on the T‐cells should be verified in the ovarian cancer ascites.

#### Adenosinergic pathway contributes to the immunosuppressive status in the ovarian cancer ascites

##### The general mechanism of adenosine‐mediated immunosuppression

Adenosine in the TME is mainly obtained from ATP degradation. Previous studies have demonstrated that the extracellular ATP concentration in the tumor tissue was a 1,000‐fold higher than that in the normal tissue. The superabundant ATP in the TME derives mainly from large numbers of dying cells.^73^

The extracellular ATP can be degraded into adenosine by ectonucleotidases (CD39 and CD73).^33^ These adenosines bind to the adenosine receptors, such as A1R, A2AR, A2BR and A3R to induce the downstream regulation and mediate a series of protumor and immunosuppressive effects.^33^ These adenosine receptors, CD39 and CD73, which are termed as “adenosinergic molecules,” are expressed on the plasma membrane of various cell types, including the tumor, stromal and immune cells.^33^ Adenosine can directly or indirectly suppress the T‐cell function. For example, the activation of A2AR induces the differentiation of CD4^+^ T‐cells into Tregs,^74^ while the CD39 and CD73 expressed by Tregs further strengthen the immunosuppression.^75^ Additionally, the A2BR activation on the MDSCs promotes the secretion of VEGF, which indirectly suppresses T‐cell function.^76^

Hypoxia, a characteristic of TME, promotes the immunosuppressive effects of the adenosinergic pathway. Hypoxia can promote ATP release, which provides raw materials for the adenosine synthesis.^73^ Hypoxia can also activate HIF1α to promote the expression of CD39, CD73 and A2BR, which strengths the adenosinergic pathway.^33^ Additionally, the levels of adenosinergic molecules are also influenced by cytokines (such as TNFα and TGFβ) in the TME.^33^ Thus, the level of adenosinergic molecules is closely related to the overall immune status of TME.

##### Adenosinergic pathway is closely related to the immunosuppressive status of the ovarian cancer microenvironment

Many studies have demonstrated that the adenosinergic pathway contributes to immunosuppression in the TME of ovarian cancer.^77^ Clinically, the enhanced expression level of CD73 in the tumor tissue predicts a poor prognosis in patients with ovarian carcinoma.^78^ The tumor cells from the ovarian cancer ascites express functional CD39 and CD73, which promote the generation of adenosine.^79^ Moreover, the macrophages derived from the malignant ascites exhibit enhanced expression of CD39 and CD73.^56^ These results suggest that the adenosinergic pathway is activated in the malignant ascites.

Further studies are needed to assess the contribution of the adenosinergic pathway in promoting immunosuppression in the malignant ascites. Interestingly, adenosine cannot be detected in the cell culture supernatant of ovarian tumor cells by high‐performance liquid chromatography (HPLC). However, adenosine can be detected by a luciferase‐based assay employing the ADORA2A‐transfected HEK‐293 “sensor” cells.^79^ These results suggest that the cells that exhibit CD39 and CD73 expression may produce a high adenosine level in the local region. However, the newly generated adenosine may be rapidly diluted or degraded in the microenvironment. Thus, adenosine in the malignant ascites may be maintained at a high level in the local region, which modulates the antitumor immunity *via* a paracrine/autocrine mechanism.

#### High level of ROS suppresses T‐cell function in the malignant ascites

##### Ovarian cancer ascites exhibits enhanced ROS level

The ovarian cancer ascites exhibits enhanced levels of ROS.^6^, ^70^, ^75^ Hypoxia, robust glycolysis and lipid metabolism contributes to ROS production in the malignant ascites and adjacent solid tumors.^32^ The sources of ROS are tumor cells, CAFs, Tregs, MDSCs and immune effector cells. H_2_O_2_ is the most reactive form of ROS, which can be released into the microenvironment and promote signaling at long‐distance.^32^ The ROS derived from the tumor cells can promote glycolysis in CAFs, and the enhanced glycolysis may further increase ROS production.^32^

##### The immunosuppressive effects induced by ROS

In addition to oxidative stress, ROS mediates multiple effects. An appropriate level of ROS benefits T‐cell functioning. However, excessive ROS levels can directly impair the T‐cell function,^80^ which can disturb the redox balance of T‐cells and inhibit T‐cell activation.^81^, ^82^ The high level of ROS in the ovarian cancer ascites can inhibit the T‐cell function. Previous studies have demonstrated that the level of ROS in the CD4^+^ T‐cells increases after *in vitro* treatment with the cell‐free ascites.^6^ Additionally, incubation with the cell‐free ascites also enhances the ROS level in DCs, which induces ER stress and subsequently impairs the antigen‐presenting capacity of DCs and inhibits T‐cell activation.^70^ Moreover, the MDSCs and Tregs accumulate in the ovarian cancer ascites. These cells can release ROS molecules to suppress effector T‐cells.^20^, ^83^

## Summary and Perspective

**Figure 3:**
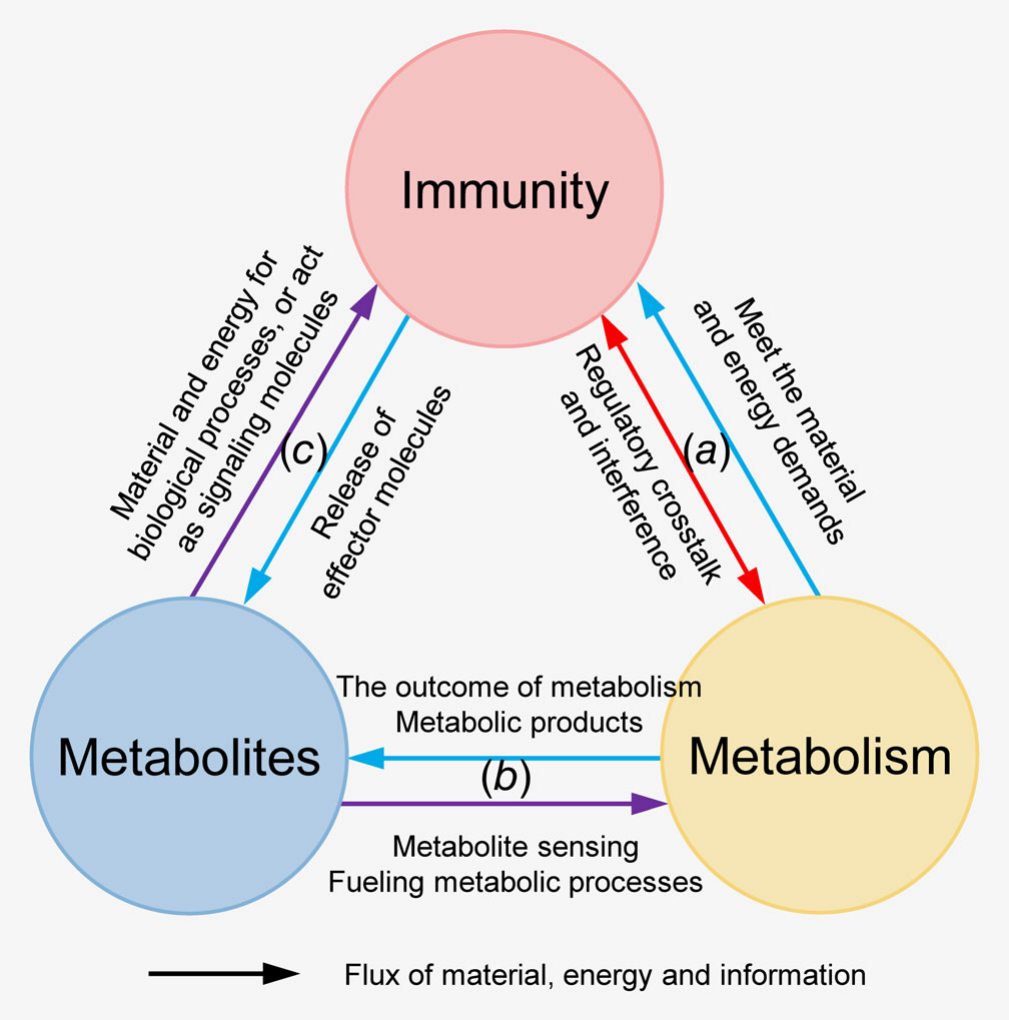
Metabolic factors are closely linked to immune functions. (*a*) The metabolic process provides energy and materials for the immune process. The regulatory pathways of metabolic and immune processes are highly coupled to each other. (*b*) The composition and levels of intracellular and extracellular metabolites can reflect and directly affect the metabolic status of the cells. Simultaneous metabolomic analysis of large numbers of metabolites can be useful for determining the metabolic status of cells. (*c*) Some types of metabolites are closely related to immunity. Some metabolites (such as nitric oxide) are directly involved in the immune processes by functioning as signaling and effector molecules. Additionally, the levels of amino acids, such as tryptophan and arginine, can markedly affect the functions of immune cells.

The immune status of TME is determined by various factors. Recently, several metabolic factors in the TME were reported to be the key factors involved in immunosuppression. This is because metabolism provides energy and materials for cell growth and function, and the regulatory pathways of metabolism are coupled to the global regulation network (Fig. 3). Although several common metabolic mechanisms of immunosuppression have been elucidated in different types of cancer, it is important to study the characteristics and mechanisms of immunosuppression in a specific cancer type. In this review, we have summarized the metabolic factors in the microenvironment of ovarian cancer, especially those that can affect the immune status of the malignant ascites microenvironment.

Ovarian cancer is located in the abdominal cavity and is often associated with the formation of ascites. *There is an exchange of materials between the ascites and solid tumor tissues and between the ascites and circulatory system through the peritoneum* (Fig. 1). Therefore, the ovarian cancer TME should be considered as a whole. The exchange of materials plays an important role in shaping the ovarian cancer microenvironment *via* various metabolites and other factors, such as cytokines, chemokines and so on, which influence the immune status profoundly.

The metabolic features of the ovarian cancer TME may be predominantly determined by the metabolism of tumor cells and tumor‐associated stromal cells. Large numbers of tumor cells undergo the Warburg effect to meet their energy and material demands. Actually, reverse Warburg effect cooperates with Warburg effect in TME.^36^, ^37^ For example, the invasive tumor cells are reported to exhibit robust oxidative phosphorylation fueled by reverse Warburg effect.^36^ First, cancer cells induce the aerobic glycolysis in neighboring stromal fibroblasts. Then, these CAFs secrete lactate and pyruvate. As a result, cancer cells take up these energy‐rich metabolites and facilitate mitochondrial TCA cycle, thereby promoting efficient energy production, resulting in a higher proliferative capacity. Furthermore, the adipocytes in omentum may supply abundant lipids to the microenvironment of ovarian cancer,^59^ which is a distinctive and unique feature of ovarian cancer microenvironment. These metabolic processes mentioned above together contribute to low oxygen, lack of nutrients and accumulation of metabolites in the microenvironment of ascites.

The metabolic factors, such as hypoxia, nutrient deficiency and accumulated metabolic products, have multiple effects on the intracellular metabolic sensors and regulators (such as HIF1α and PPARs). Consequently, they can not only affect the metabolic processes, but also modulate the immune regulation, as well as the global signaling network of various types of cells. It is worth noting that the metabolic factors in the solid tumor tissue may affect the immune status of the malignant ascites. Hypoxia, as well as soluble factors induced by hypoxia, can mediate immunosuppression through multiple mechanisms (Fig. 2). Additionally, the abundant lipids in the ovarian cancer microenvironment regulate immune functions in a variety of ways. In summary, *hypoxia, lipid enrichment and their relevant regulators play a comprehensive and critical role in shaping the immune status of the ovarian cancer microenvironment in both intracellular and extracellular manner*.

Future studies must elucidate the mechanism underlying the interactions between immune and metabolic responses in the TME of ovarian cancer.

### Future studies on elucidating the mechanisms

In addition to the metabolites in the ascites, other factors, which is directly related to the metabolic process and regulation, are also considered as metabolic factors. These factors (such as HIF1α, GLUT1, ARG1, PPARs, CD39, etc.) include various metabolic enzymes, metabolite transporters, sensors, as well as signal transductors. They participate in the regulation of T‐cell function holistically. The interactions between the metabolites and other metabolic factors need further elucidation, which can aid in a better understanding of the mechanisms underlying the metabolite sensing, and the role of various metabolites in the ascites. Additionally, newly discovered mechanisms need to be verified in the ascites. For example, ferroptosis is an iron‐dependent, nonapoptosis form of cell death, which represents the dysregulation of iron metabolism and lipid peroxidation.^84^ Indeed, lipid peroxide accumulation is observed in some types of immune cells in the malignant ascites. Future studies should identify the potential factors in the malignant ascites that trigger or inhibit ferroptosis, and investigate their influence on the survival of various cells in the TME.^85^, ^86^

### Potential clinical transformation

Ovarian cancer has the following characteristics: a high tendency of metastasis, inherent chemo‐resistance, inefficiency in immunotherapy.^87^, ^88^, ^89^ The ovarian cancer ascites is reported to promote tumor progression and metastasis and inhibit tumor cell death induced by immune cells or chemotherapy.^10^ Thus, there is an urgent need to explore the correlation between the specific metabolites in the ascites and response to chemotherapy or immunotherapy. Additionally, it is important to further analyze the differences in the metabolic factors between the different histologic types of ovarian cancer. These studies may provide novel biomarkers for diagnosis and prognosis, as well as new targets for drug development.

### Ongoing technological innovation

To fully understand the metabolic features of TME, it is necessary to develop real‐time, dynamic and visualized metabolite detection technology to monitor the level of various metabolites inside and outside the cells. Currently, various new technologies and strategies, including positron emission tomography (PET), magnetic resonance imaging (MRI), mass spectrometry imaging (MSI) and some genetically encoded metabolite sensors, are being continuously developed to understand the metabolic features of the TME.^90^, ^91^, ^92^, ^93^

The ascites, a pathological effusion, are easier to obtain than solid tumor tissue and extracellular fluid in tissues. Compared to the peripheral blood, the ascites is more powerful to reflect the features of ovarian cancer. Thus, the ascites is an ideal model to study the TME, especially the extracellular environment. The study of metabolic factors in the malignant ascites can aid in elucidating the mechanism underlying the occurrence and development of ovarian cancer and provide new clues for diagnosis and treatment.

## Conflict of interest

The authors declare no conflicts of interest.
